# Syntax-like Structures in Maternal Contact Calls of Chestnut-Crowned Babblers (*Pomatostomus ruficeps*)

**DOI:** 10.1007/s10764-022-00332-9

**Published:** 2022-11-05

**Authors:** Silvan Spiess, Helen K. Mylne, Sabrina Engesser, Joseph G. Mine, Louis G. O’Neill, Andrew F. Russell, Simon W. Townsend

**Affiliations:** 1 Comparative Communication and Cognition Group, Department of Comparative Language Science, University of Zürich, Affolternstrasse 56, Zurich-Oerlikon, 8050 Switzerland; 2 Center for the Interdisciplinary Study of Language Evolution (ISLE), University of Zürich, Affolternstrasse 56, Zurich-Oerlikon, 8050 Switzerland; 3 Centre for Ecology & Conservation, University of Exeter, Cornwall Campus, Penryn, TR10 9FE Cornwall UK; 4 Department of Behavioral & Cognitive Biology, University of Vienna, Djerassi-Platz 1, 1030 Vienna, Austria; 5 Fowlers Gap Arid Zone Research Station, School of Biological, Environmental and Earth Sciences, University of New South Wales, Fowlers Gap, via Broken Hill, NSW 2880 Australia; 6 Department of Biological Sciences, Macquarie University, 14 Eastern Road, Sydney, NSW 2109 Australia; 7 Department of Psychology, University of Warwick, University Road, Coventry, CV4 7AL UK

**Keywords:** Combinatoriality, Vocal communication, Language evolution, Syntax

## Abstract

The combination of meaning-bearing units (e.g., *words*) into higher-order structures (e.g., compound words and phrases) is integral to human language. Despite this central role of syntax in language, little is known about its evolutionary progression. Comparative data using animal communication systems offer potential insights, but only a handful of species have been identified to combine meaningful calls together into larger signals. We investigated a candidate for syntax-like structure in the highly social chestnut-crowned babbler (*Pomatostomus ruficeps*). Using a combination of behavioral observations, acoustic analyses, and playback experiments, we test whether the form and function of maternal contact calls is modified by combining the core “piping” elements of such calls with at least one other call element or call. Results from the acoustic analyses (236 analysed calls from 10 individuals) suggested that piping call elements can be flexibly initiated with either “peow*”* elements from middle-distance contact calls or adult “begging” calls to form “peow-pipe” and “beg-pipe” calls. Behavioral responses to playbacks (20 trials to 7 groups) of natural peow-pipe and beg-pipe calls were comparable to those of artificially generated versions of each call using peow elements and begging calls from other contexts. Furthermore, responses to playbacks (34 trials to 7 groups) of the three forms of maternal contact calls (piping alone, peow-pipe, beg-pipe) differed. Together these data suggest that meaning encoded in piping calls is modified by combining such calls with begging calls or peow elements used in other contexts and so provide rare empirical evidence for syntactic-like structuring in a nonhuman animal.

## Introduction

Language is unique to humans and integral to our ecological success, but its origins remain an enigma. For example, some scholars advocate that the capacity for full-blown, hierarchical syntax emerged suddenly during hominin evolution (Berwick & Chomsky, 2019), whereas others suggest that it evolved gradually from early rudiments (Martins & Boeckx, 2020). In its most basic structural form, syntax involves combining at least two meaning-bearing units to form a new meaningful sequence. Such concatenated structures can arise in at least one of three main ways (Collier *et al*., 2014; Townsend *et al*., 2018). First, under predicate argument structure, the meaning of the predicate (e.g., action) is complemented by the information regarding signaler identity (e.g., “*I (Bob*) *move*”). Second, under modification, a meaningful stem word (e.g., “*quick*”) is combined with sound units carrying a more abstract meaning (e.g., “-*ly*”) to form a compound word (i.e., “*quick-ly*”). Third, two stem words, both bearing meaning in isolation, can be added together, either to generate compositional meaning as in a basic conjunction (e.g., as in “*come [and] fight*”) or a wholly new meaning, as in idiomatic compounds (e.g., “*cold feet*” meaning nervous). Given that vocal combinations have been proposed to evolve when the efficiency of information transfer is enhanced by building on existing signals rather than generating new ones (Nowak & Krakauer, 1999; Nowak *et al*., 2002) and that the need for increased information likely correlates with social complexity (Freeberg *et al*., 2012; Leighton, 2017; Leighton & Birmingham, 2021; Peckre *et al*., 2019), the study of call combinations in social animals might offer insights into the origin of syntax-like communication processes and its early forms (Collier *et al*., 2017).

Although evidence for hierarchical syntactic structures is lacking outside of human language, an emerging body of literature in mammals and birds suggests that vocal repertoires can be modified by combining calls and/or call segments into compound structures—with ostensible analogues to rudimentary syntactic processes (Engesser & Townsend, 2019; Leroux & Townsend, 2020). Studies in a handful of species are particularly noteworthy, because they confirm through acoustic analyses and/or playback experiments that the stem calls in compounds are equivalent to those used in isolation (and therefore are unambiguously meaning-bearing). For example, Diana monkeys (*Cercopithecus diana*; Candiotti *et al*., 2012) and banded mongoose (*Mungos mungo*; Jansen *et al*., 2012) concatenate calls cueing individual identity with call or call-segments associated with social events (socio-positive or -negative) or behavior (foraging, moving, searching), respectively. This is suggested to be akin to a rudimentary predicate argument structure, wherein the signaler exposes its identity in combination with its current state (Collier *et al*., 2014). Campbell’s monkeys (*C. campbelli*) can temper the urgent “kraak” alarm calls with an “oo” suffix; the latter not used in isolation and so functioning as an affixation-like entity (Coye *et al*., 2015; Ouattara *et al*., 2009; Schlenker *et al*., 2014, 2016). Similarly, pied babblers (*Turdoides bicolor*) can modify their recruitment “A” calls by suffixing such calls with “B” sound elements which serve to modify the form of recruitment from approaching to following the signaler (Engesser *et al*., 2018). By contrast, putty-nosed monkeys (*C. nictitans*) combine two calls independently used in alarm contexts to generate a third call structure that initiates a qualitatively new movement response, suggestive of an idiomatic structure (Arnold & Zuberbühler, 2006). Finally, Japanese tits (*Parus minor*) and pied babblers combine alarm and recruitment calls to induce group-level mobbing, both of which are suggestive of conjunction-like compositionality (Engesser *et al*., 2016; Suzuki *et al*., 2016). The take-home messages from such studies are that: (a) a broad range of proto syntactic-like processes have been uncovered in studies of social animals; but (b) more studies are clearly required to elucidate whether certain forms predominate and under what circumstances.

**Fig. 1:**
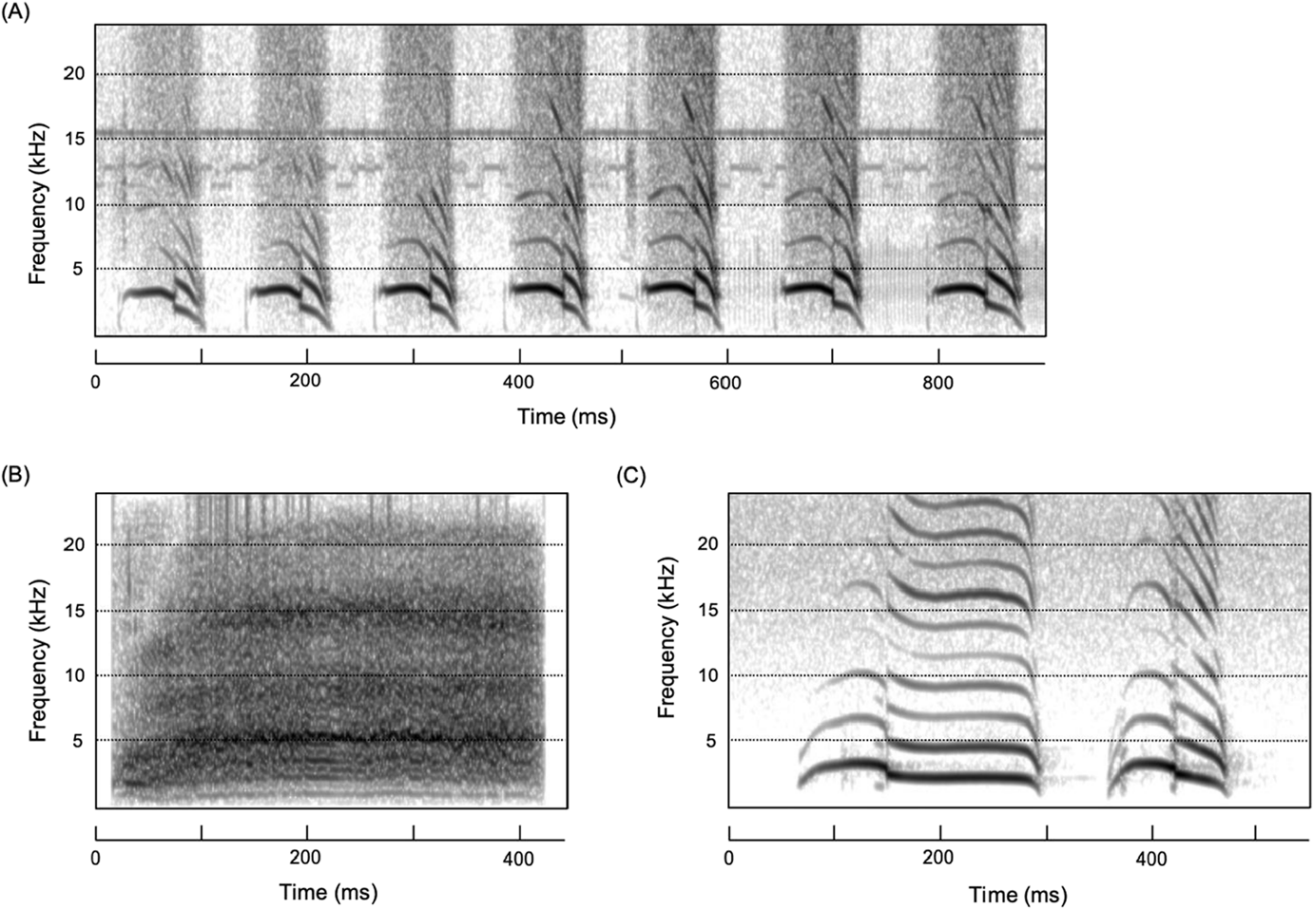
Spectrograms of the three key calls featured in common variants of chestnut-crowned babbler maternal contact calls, recorded at Fowlers Gap, New South Wales, AUS in the breeding season of 2017. (**A**) Piping call representing the defining stem of all maternal contact calls, being characterized by a series of loud, high-pitched “piping” elements audible over several hundred meters; (**B**) adult begging call showing typical broadband structure, often overlaying varying extents of harmonic structures, used during allo-feeding; and (**C**) middle distance (peow-pee) contact calls, which serve to maintain contact and recruit group members together over several tens of meters.

Anecdotal observations suggest that the chestnut-crowned babbler (*Pomatostomus ruficeps*) from inland southeastern Australia also might incorporate syntax-like structures in its communication system. Like many other group-living birds, this 50 g cooperative breeder has a rich vocal repertoire of at least 18 functionally distinct calls (Crane *et al*., 2016). Of these, the maternal contact call is particularly noteworthy for at least two reasons. First, it is only produced by the dominant females of groups—the only female individuals to reproduce, and only during the breeding season—a restricted time period from the weeks before egg-laying until nestling fledging. This call appears to play a role in recruiting partners and helpers to potential breeding opportunities and coordinating care at the nest following egg-laying and particularly hatching (Crane *et al*., 2016). Second, although the quintessential stem of maternal contact calls comprise a series of strident, high-pitched “piping” elements (Fig. 1A), anecdotally, such calls are often preceded by other independent calls and/or call elements. Most notably, “piping” calls can be initiated by calls that appear reminiscent of adult “begging” calls used by females in association with allo-feeding by other group members (Fig. 1B) or by the first (“peow”) element of middle-distance contact (“peow-pee”) calls that are used in recruitment and group cohesion (Fig. 1C) (Crane *et al*., 2016). These observations suggest that maternal contact calls offer a candidate syntactic-like structure, providing further insights into the form of syntactic-like processes in nonhuman animals.

Broadly, our objectives are to investigate the case for syntax-like structures in the maternal contact calls of chestnut-crowned babblers and to elucidate the forms and potential functions of such combinatoriality using acoustic analyses and playback experiments. To these ends, we have the following three goals. First, we compare the acoustic properties of the three call elements most commonly found in maternal contact calls (i.e., the stem-piping call elements, begging call elements, and peow elements from peow pee calls) to ensure each is acoustically distinct, and then test responses to playbacks of each call to ensure that they induce measurable differences in behavioral response. Second, we test whether the begging and peow elements that often are apparently combined with the stem piping elements in maternal contact calls are acoustically and perceptibly equivalent to each call element produced independently in begging and peow pee (middle-distance) contact calls, respectively. In this case, we: (a) compare the acoustic structures of begging and peow elements in combinations with piping elements in maternal contact calls versus their structures in begging and middle-distance contact call contexts; and (b) compare behavioral responses to playbacks of natural maternal contact calls initiated by begging or peow elements and artificial ones in which begging or peow elements from these other contexts are appended to the start of piping calls naturally lacking such elements. Finally, we use playbacks to test whether the three forms of maternal contact call combinations under consideration here (i.e., piping calls alone, beg-pipes, peow-pipes) generate differential behavioral responses, the final criteria for syntax-like structuring.

## Methods

We performed our study on a color-ringed population of wild chestnut-crowned babblers that has been monitored since 2004 at Fowlers Gap Arid Zone Research Station in the arid zone of New South Wales, Australia (141°39’E, 31°06’S) (Russell, 2016). The habitat is dominated by low, open shrubland with tall shrubs and trees in which babblers nest restricted largely to short linear stands in creeks and drainage lines. As a consequence, few obstacles can interfere with sound integrity in this open arid landscape.

### Acoustic Evidence for Syntax-like Combinatoriality in Maternal Contact Calls

To assess the evidence for combinatoriality in maternal contact calls and elucidate their potential combinatorial structure and function, we obtained audio tracks from video-files recorded using a small endoscopic MO-S408 camera with in-built audio capacity (Misumi Electronics Corporation; sampling rate = 48 kHz, bit-depth = 32) placed inside babbler nests. We converted the audio lines from the videos into WAV files to process the spectrograms with Adobe Audition CS 6 (Version 5.0) and discarded files with excessive background noise or interference, mainly by wind and overlapping calls. The video files included varied in duration from 50 min to 2 h. To ensure an even distribution of sampling of calls from each sound file, we divided files into 5 intervals of equal duration and extracted the first 10 maternal contact calls in each interval, although in some cases we extracted additional calls from other intervals if some intervals contained less than 10 calls. We aimed for a total of 50 maternal contact calls per audio file and discarded files containing less than 30 calls. Overall, we extracted 273 maternal contact calls from 10 breeding females. From visual inspection of these calls, and in line with anecdotal observations, it was apparent that maternal contact calls often contain elements in addition to the core piping elements. We inspected the spectrogram of each of these apparently additional elements in each call visually against a library of other babbler calls (Crane *et al*., 2016) to identify candidate syntactic-like structures.

In addition to the core piping elements in each maternal contact call, we identified at least five other calls or call elements produced in association with maternal contact calls (Table I), but we excluded three of these for the purposes of this study. Those excluded were maternal contact calls, including: (a) repeat-element alert calls that can precede maternal contact calls but are currently of unassigned function and are ambiguously part of the maternal contact call; (b) repeat-element long-distance contact call elements that often are embedded within maternal contact calls, but whether their inclusion constitutes a call change, albeit within maternal contact call sequences, or part of the maternal contact call is unclear; and (c) pee elements (from middle-distance, “peow pee” calls that only rarely (< 5%) precede piping elements. We thus concentrate on the two most common and strongest candidates for combination calls within the maternal call complex (N = 187 of 236 calls). In the here-named “peow-pipe,” piping elements appear to be initiated by a peow element found in middle-distance contact (“peow-pee”) calls (Fig. 1C; Table I), and in the here-named “beg-pipe,” the piping elements appear to be initiated by a broadband adult begging call (Fig. 1B; Table I). To elucidate the case for combinatoriality in these two variants of maternal contact calls, we extracted up to 16 spectral parameters relating to fundamental frequency, duration, and energy distribution of 148 peow elements from 148 peow-pipe calls, 39 begging elements from 39 beg-pipe calls and 408 pipe elements taken from all 236 maternal calls. In addition, we extracted the same spectral parameters from 40 peow elements from 40 middle-distance contact calls and 40 begging calls chosen at random from our library (Table II). For begging calls, we were only able to extract parameters relating to duration and energy distribution, because these broadband call elements typically lack a clear fundamental frequency (Table II).

**Table I:** Call types, basic structure and context or function of relevant chestnut-crowned babbler calls (Crane *et al*., 2016)

Call type	Basic structure	Context/function
Maternal contact call	Series of strident piping elements often preceded by other calls or call elements	Exclusively produced by females before/during breeding
Piping call	Maternal contact call comprised entirely of piping elements	Signals female location during breeding
Begging call	Broadband call repeated 1–3 times	Produced during allo-feeding
Middle-distance contact call	Typically bi-element call of form “peow-pee,” although “pee” elements can be repeated 2–4 times	Used to facilitate recruitment and group cohesion over
Beg-pipe	Maternal contact call initiated by begging call	Typically used in context of allo-feeding
Peow-pipe	Maternal contact call initiated by the first (“peow”) element of middle-distance contact calls	Thought to be used to recruit group members to female or nest
Alert call	Series of loud repeated “chow” elements	Function unclear: commonly precedes maternal contact calls
Long-distance contact call	Series of loud 3–8 repeated elements	Produced when individuals get separated from the group and to locate group members
Beg-squawk	Begging call followed by several loud “squawks” with both tonal and broadband properties	Overt call used to stimulate allo-feeding used in-association with wing fluttering

**Table II:** Sixteen spectral parameters extracted from chestnut-crowned babbler recordings collected in Fowlers, New South Wales, AUS in the breeding season of 2017, and used in discriminant function analyses. We extracted parameters using an automated script developed in Praat v.5.0.47 (Briefer 2012; Briefer *et al*., 2015; Watson *et al*., 2018). Because broadband calls lack a clear fundamental frequency, we were only able to use sound duration, energy quartiles, % time of max intensity, amplitude variation and shimmer for begging calls, and beg elements from beg-pipes

Parameter	Description
Sound duration	Duration of the acoustic element in seconds
Mean F0	Mean fundamental frequency of the acoustic element in Hertz
Starting F0	Fundamental frequency at the start of the acoustic element in Hertz
Ending F0	Fundamental frequency at the end of the acoustic element in Hertz
Maximum F0	Maximum fundamental frequency across the acoustic element in Hertz
% time of max. F0	Percentage of the total element duration when the fundamental frequency reaches a maximum
F0 absolute slope	Mean absolute slope (steepness) of the fundamental frequency across the acoustic element
F0 variation	Mean fundamental frequency variation per second, calculated as the cumulative variation in the fundamental frequency contour in Hertz divided by the element duration
Peak frequency	Component frequency with the highest power/energy of the acoustic element
25% energy quartile	Frequency values at the upper limit of the first quartile of energy measured on a linear amplitude spectrum applied to the whole acoustic element
50% energy quartile	Frequency values at the upper limit of the second quartile of energy measured on a linear amplitude spectrum applied to the whole acoustic element
75% energy quartile	Frequency values at the upper limit of the third quartile of energy measured on a linear amplitude spectrum applied to the whole acoustic element
% time of max intensity	Percentage of the total acoustic element when the intensity reached a maximum
Amplitude variation	Mean amplitude variation per second, calculated as the cumulative variation in amplitude divided by the element duration
Jitter	Mean absolute difference between frequencies of two consecutive fundamental frequency periods, divided by the mean frequency
Shimmer	Mean absolute difference between amplitudes of two consecutive fundamental frequency periods, divided by the mean amplitude

### Playback Experiments

We performed 62 playbacks on seven groups of wild babblers in the field (Table III). The number of groups used was limited due to recent droughts and the difficulty of reliably finding birds during the experimental phase of this study. We performed experiments in random order on each group and no group received the same playback sets. First, we performed 34 playbacks of natural begging calls (N = 15 trials), middle-distance contact calls (N = 13 trials), and piping calls (N = 14 trials) to determine baseline responses to each and ensure that each generates distinct behavioral responses (Aim 1). Second, we performed 20 natural and artificial beg-pipe and peow-pipe playbacks to test whether responses were functionally comparable between natural and artificially generated versions of beg-pipes and peow-pipes (Aim 2). This layer of the experiment is required to test whether the call or call elements used in other contexts are perceptibly equivalent when used in combinations with the core piping elements. In this case, we contrasted responses to natural beg-pipe (N = 5 trials) and peow-pipe (N = 5 trials) playbacks against responses to artificial versions of each, wherein we generated artificial beg-pipe and peow-pipe calls by substituting the apparent begging and peow elements with those occurring naturally outside of maternal call contexts (N = 5 playback trials each). Finally, we contrasted responses to the three variants of maternal contact calls (piping element only, beg-pipes, and peow-pipes) to elucidate the function of combinatoriality in this call complex.

**Table III:** Summary of playback trials, performed on chestnut-crowned babbler groups in Fowlers Gap, New South Wales, AUS in the breeding season of 2019. We varied the composition of recordings in playbacks, such that no playbacks included the same sound elements. No group received the same playback call type on the same day

Aim	Playback set	Purpose	Sample size
Aim 1	Natural piping, middle-distance contact (peow-pee) & begging calls	Determine baseline behavioral responses to each call & ensure that playbacks of each generate distinct responses	Piping: 14 trials to 7 groups Peow-pee: 13 trials to 7 groups Begging: 15 trials to 7 groups
Aim 2	Natural beg-pipes & peow-pipes vs. artificial beg-pipes & peow-pipes	Test whether call or call elements used in other contexts are perceptibly equivalent when used in combinations with the core piping elements – paramount to defining combinatoriality	Nat. beg-pipes: 5 trials to 5 groups Nat. peow-pipes: 5 trials to 5 groups Art. beg-pipes: 6 trials to 6 groups Art. beg-pipes: 4 trials to 4 groups
Aim 3	Piping calls vs beg-pipes & peow-pipes	Investigate whether in beg-pipes and peow-pipes concatenation of elements/calls serves to modify meaning	Piping: 14 trials to 7 groups Beg-pipes: 10 trials to 7 groups Peow-pipes: 10 trials to 7 groups

Playback sets to each group on each day consisted of piping calls, begging calls, peow pee (middle distance) calls, beg-pipes (natural or artificial), and peow-pipes (natural or artificial). We performed playbacks in up to three sessions in each group (2–60 d between sessions, mean = 11 d), and never played back more than five trials per session to reduce habituation effects. A minimum of 10 min separated successive trials on the same day, after which birds returned to normal behavior and moved > 50 m away. We generated sufficient playback sets to ensure that no call was played back more than once to any group, and, in each session, we played call types in random order. No groups received calls recorded from the same or a neighboring group to remove effects of expectancy violation or familiarity (Crane *et al*., 2015). Finally, we broadcast all playbacks from a concealed position within the center of a group’s home range using a Braven BRV-X speaker, connected via an AUX-cable to a smartphone (Nokia 6, 2017) 5 m from the speaker (sampling rate = 48 kHz, bit-depth = 32).

We created playback tracks from recordings made at Fowlers Gap in 2017 using Adobe Audition. When we generated artificial maternal contact call stimuli (combinations of pipe elements from MCCs and in isolation produced begging calls or peow elements from peow-pees), we normalized them by ensuring that the relative difference in amplitude between the component parts matched the amplitude differences found between elements of natural MCCs. We played back each type of stimulus in line with its naturally occurring amplitude, and we adjusted it by ear. All stimuli were played back at the same volume settings on the speaker. For stimuli comprising piping elements, which naturally occur with a variable number of repetitions, the number of piping elements was kept constant between stimuli to the same group (5–7 element repetitions). We kept intervals between the calls in artificially created maternal contact calls at 20 ms, the mean natural interelement interval between the maternal contact calls’ starting element (beg or peow) and the subsequent piping elements (this study, N = 60 calls). Each treatment track consisted of ten repetitions of the same stimulus with breaks of 2 s between them, being in line with the natural production of the call. We recorded responses with a compact video camera (Sony Handycam HDR-CX240). We coded the videos using BORIS v. 7.7.3. Specifically we noted vocal responses to playbacks given the highly salient nature of this behavioral variable. All vocalizations (Table I) were recorded for 1 min from the onset of the experiment.

### Statistical Analyses

We conducted Statistical analyses in R (version 3.5.3). We used cross-validated discriminant function analyses (DFA, lda function from MASS package, Venables & Ripley, 2002) to three ends. First, we verified that piping and peow elements are acoustically distinct (begging elements are obviously distinct being broadband) (Aim 1). In these analyses, we compared the extracted acoustic parameters (Table I) pertaining to fundamental frequency, duration, and energy distribution of a single randomly selected peow and piping elements from each of 10 maternal contact calls (N = 10 females). Second, we used the same approach to test whether peow elements in maternal contact calls (N = 10 individuals) are discernible from peow elements in middle-distance contact calls (N = 10 individuals) as well as whether begging elements produced in maternal contact calls (N = 10 individuals) are discernible from those produced alone (N = 10 individuals) (Aim 2). Because DFA cannot control for repeated measurements, only one call element per call type per individual was chosen at random (first sample per element type and individual) and included. Third, we used a DFA to investigate the capacity for begging, peow or piping elements to encode individual identity (as predicted under predicate argument structure) (Aim 3). In this case, multiple measures from the same individual (min. N = 2, max. N = 118, mean N = 22) were necessary to test whether each call element could be attributed to the correct individual based on acoustic parameters (begging elements from MCCs: 34 elements from 6 individuals; peow elements from MCCs: 146 elements from 9 individuals; piping: 323 piping elements from 7 females). In all analyses, to rule out correlation among the acoustic parameters, we only included parameters with a variance inflation factor lower than 10 (Fox & Weisberg, 2011). We used two-tailed, binomial tests to calculate the significance of the classification of the DFAs with a probability level depending on the number of classes discriminated.

Finally, we used a series of contingency chi-square tests and Fisher-exact tests to compare behavioral responses to playbacks (Aims 1–3). We used Fisher exact tests when assumptions of contingency tables were violated (e.g., zero observations or too many cells with expected values < 5).

## Ethical Note

All chestnut-crowned babbler research has been conducted with approvals provided by UNSW Animal Care and Ethics Committee (06/40A), Macquarie University, The University of Exeter, NSW National Parks and Wildlife Service and the Australian Bird and Bat Banding Scheme (3340). This work was conducted on the land of the Barkandji clan of the Paakantyi nation.

## Results

### Structure of and Responses to Constituent Components of Maternal Contact Calls (Aim 1)

**Fig. 2:**
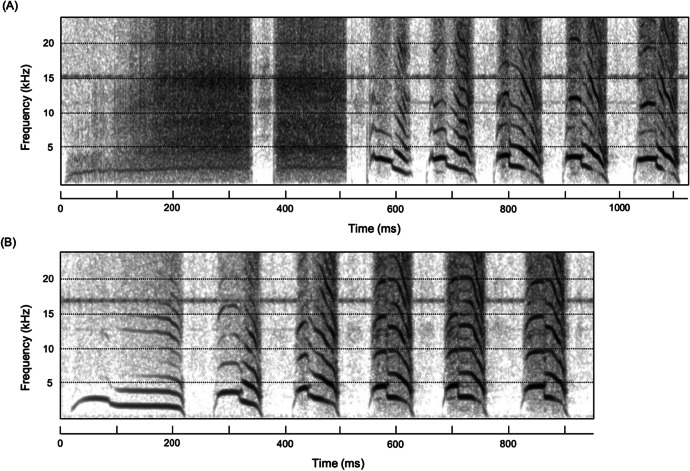
Spectrograms of two common forms of maternal contact calls found in chestnut-crowned babblers, recorded at Fowlers Gap, New South Wales, AUS in the breeding season of 2017. (**A**) Beg-pipes, with broad band begging calls preceding the piping call. (**B**) Peow-pipes, with piping calls preceded by the first (peow) element of middle-distance contact calls.

The core elements of maternal contact calls are a series of typically 5–20 high-pitched, strident, piping elements that can be produced alone (Fig. 1A) or in combinations with other calls or call elements (Fig. 2). Piping elements have a mean starting fundamental frequency of ~ 3475 Hz (± 518 SD, N = 408 piping elements from 10 groups), which rises to a maximum fundamental frequency of ~ 3,809 Hz (± 327 SD) reached ~ 31% (± 16% SD) of the way through the element, and an ending fundamental frequency of ~ 2,381 Hz (± 551 SD) approximately 0.10 s (± 0.021 SD) later. In 79% of those maternal contact calls included in this study (i.e., 187 of 236), piping elements were initiated by one of two other sound elements with strong resemblances to begging calls (21% of these cases; Fig. 2A) and peow elements (the first element in middle distance contact calls; 79% of these cases; Fig. 2B). Begging calls comprise sequences of 1–3 broadband elements of a mean duration of 0.48 s (± 0.17 SD) (N = 40 elements from 40 calls from 10 groups), whereas peow elements in middle distance contact calls have starting fundamental frequencies of ~ 2,418 Hz (± 247 SD, N = 40 elements from 40 calls from 11 groups)*,* maximum fundamental frequencies of ~ 2,824 Hz (± 196 SD), which is reached ~ 27% (± 8 SD) of the way through the element and ending fundamental frequencies of ~ 1632 Hz (± 306 SD) after ~ 0.22 s (± 0.050 SD). Being broadband, begging elements are obviously distinct from piping and peow elements, but importantly, DFA shows these latter two elements are discriminated with a success rate of 100% (against expected of 50%); at least partly because peow elements are, on average, double the length and ~ 25% lower in frequency than piping elements (DFA: N_individuals_ = 20; N_calls_ = 20; *P* < 0.0001).

**Fig. 3:**
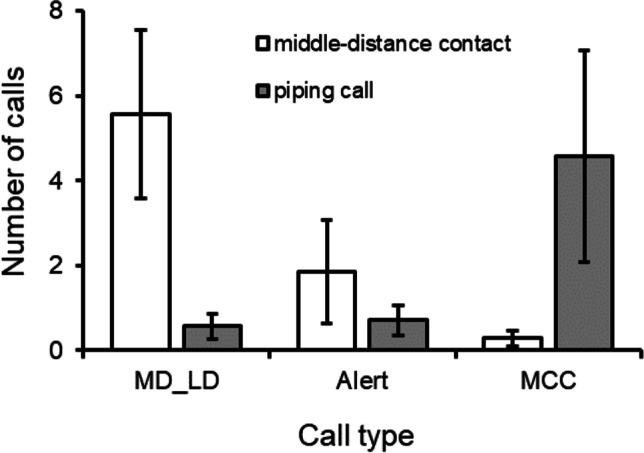
Vocal responses of chestnut-crowned babblers to playbacks of middle-distance (peow pee) contact calls and piping calls, performed at Fowlers Gap, New South Wales; AUS in the breeding season of 2019. Shows mean number of each call type produced per groups in response (± SE). MD_LD refers to middle- and long-distance contact calls responses, Alert refers to Alert call responses and MCC refers to maternal contact call (typically piping and peow-pipe) responses. Middle- and long-distance contact responses as well as alert call responses were primarily uttered by group members other than dominant females, while maternal contact call responses were all produced by the dominant female.

Playback experiments of natural begging calls (N = 15 trials), middle-distance calls (peow pee, N = 13 trials), and piping calls (N = 14 trials) generated largely qualitative differences in caller and call responses. First, while begging playbacks rarely induced any vocal response (7% of playbacks, i.e., 1 playback in 1 group), vocal responses were common following middle distance contact call playbacks (responses in 4 of 6 groups tested, 53% of trials) and piping call playbacks (responses in 5 of 7 groups tested, 50% of trials) (Fisher exact test: *P* = 0.01, based on trials of the 3 call types; *P* = 0.1, based on group responses to the three call types). Second, when vocal responses were recorded during middle-distance and piping call playbacks, the frequency of call types produced differed significantly (Contingency table χ^2^ = 57.8, d.f. = 2, *P* < 0.001, based on sums across trials) (Fisher exact test, *P* < 0.001, based on sums of means across groups) (Fig. 3). During middle-distance playbacks, group members produced a combined 39 middle-distance and long-distance calls, as well as 13 alert calls, but only 2 maternal contact calls were recorded by the breeding female. By contrast, during piping call playbacks, middle/long-distance calls (4 overall) and alert calls (5 overall) were rarely given by group members, whilst maternal contact calls by the dominant female were common (32 overall). Combined, these results show that begging calls seldom induced a behavioral response, whilst middle-distance contact call playbacks were largely met with middle/long-distance calls by group members and piping calls were met with maternal contact call responses by the dominant female.

### Are Maternal Contact Calls Combinatorial? (Aim 2)

Beg-pipes and peow-pipes appear to represent combination calls (Fig. 2). First, the time-intervals between both the begging element and the first piping element in beg-pipes as well as between the peow element and first piping elements in peow-pipes are comparable (or even less) than the interval among randomly selected piping elements within each call (beg-pipes: mean = 0.042 (± 0.007 SD) *vs.* mean = 0.046 (± 0.008 SD), respectively; paired-t_15_ =  − 2.30, *P* = 0.036) (peow-pipes: mean = 0.051 (± 0.008 SD) *vs.* mean = 0.054 (± 0.006 SD), respectively; paired-t_14_ =  − 2.94, *P* = 0.011). Second, as when uttered alone, begging elements in maternal contact calls are characterized by sequences of 1–3 elements of 0.24 (± 0.082 SD) ms duration (N = 39 from 8 groups). In addition, similarly to middle-distance contact calls, peow elements in maternal contact calls have starting fundamental frequencies of 2,403 Hz (± 429 SD, N = 148 calls from 10 groups), maximum fundamental frequencies of 2,887 Hz (± 335 SD), reached 26% (± 12%) of the way through the call, and ending fundamental frequencies of 1,492 Hz (± 266 SD) 0.23 (± 0.056 SD) s later. As predicted, given these similarities, DFA failed to distinguish between begging elements alone and in maternal contact calls (35% correct assignments from a probability of 50%; N_individuals_ = 20; N_calls_ = 20; *P* = 0.26) or peow elements in middle-distance contact calls versus maternal contact calls (60% correct assignments against expected of 50%; N_individuals_ = 20; N_calls_ = 20; *P* = 0.50).

**Fig. 4:**
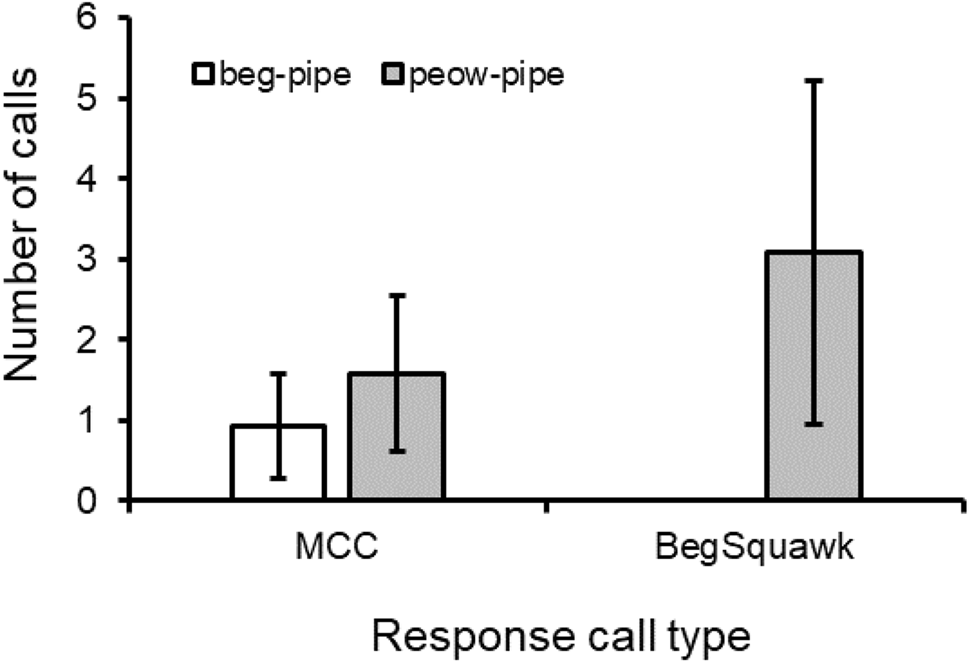
Vocal responses of chestnut-crowned babblers to playbacks of beg-pipes and peow-pipes, performed at Fowlers Gap, New South Wales; AUS in the breeding season of 2019. Mean number of maternal contact and beg squawk calls produced by dominant females in each group in response to each playback type (± SE). MCC refers to maternal contact calls (typically piping and peow-pipe responses). Only peow-pipes induced beg-squawk responses.

Furthermore, playback experiments comparing responses to natural versus artificial versions of peow-pipe and beg-pipe maternal contact calls, where we generated artificial calls by taking peow and begging elements from middle-distance and begging calls, respectively, reinforced the case for combinatoriality. Overall, five of the seven groups tested responded to such playbacks. The most common responses to these maternal contact call playbacks were maternal contact calls (30 calls, 5 groups) and beg-squawks (32 calls, 4 groups), both uttered by the dominant female in each group, and only sporadic long-distance contact calls (7 calls, 4 groups) and middle-distance contact calls (4 calls, 2 groups) were produced by other group members. There were no differences in the frequencies with which maternal contact calls (18 *vs.* 12) and beg-squawks (15 *vs.* 17) were produced in response to natural versus artificial playbacks (Chi-squared: χ^2^ = 0.61, d.f. = 1, *P* = 0.44), although only two beg-pipe trials (1 artificial and 1 natural) generated maternal contact call responses, and none generated beg-squawk responses. As a consequence, peow-pipe playbacks generated double the number of maternal contact call responses than beg-pipes (19 from 60% of trials *vs.* 9 from 18% of trials) and were the only playbacks to generate beg-squawk responses (32 from 40% of trials *vs.* 0). This led to significant differences in the responses of dominant females to beg-pipes versus peow-pipes across trials and groups (Fisher exact test: *P* = 0.001, based on sums of trials; *P* = 0.0045, based on sums of means across groups) (Fig. 4). Together, these results at least suggest that peow-pipes represent combination calls and that begging calls and the peow element from middle-distance contact calls are added to the beginning of piping calls to modify meaning.

### Potential Syntax-like Form and Function (Aim 3)

Discriminant function analyses on begging, peow and piping elements from maternal contact calls revealed significant among-female variation in acoustic parameters in all three call elements. Specifically, ~ 62% of 34 begging elements from 6 females were correctly assigned against an expected probability of 17% (Binomial test, *P* < 0.001), whereas ~ 48% of 146 peow elements were correctly assigned from 9 females against a probability of 11% (Binomial test *P* < 0.001) and 53% of 323 piping elements from 7 females were correctly assigned to a given female against a probability of 14% (Binomial test *P* < 0.001). Thus, all three elements are around 4 times more likely to be assigned to the correct female than by chance alone, meaning that all three provide similar information on individual identity; so neither the addition of begging nor peow elements appear to enhance the individuality of piping calls.

**Fig. 5:**
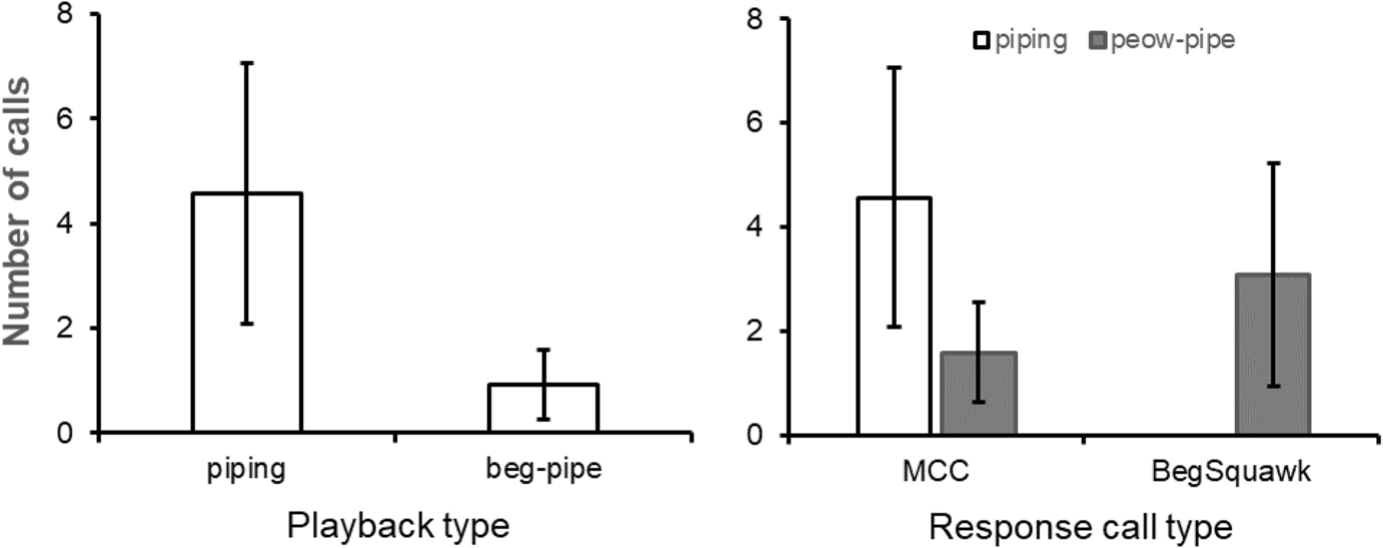
Vocal responses of chestnut-crowned babblers to playbacks of different forms of maternal contact call, performed at Fowlers Gap, New South Wales; AUS in the breeding season of 2019. (**A**) Mean number (± SE) of maternal contact call responses to playbacks of piping elements versus beg-pipes. (**B**) Mean number (± SE) of maternal contact call responses (MCC) and beg-squawk responses to playbacks of piping elements versus peow-pipes. Beg-squawk responses were not observed in response to playbacks of piping elements or beg-pipes.

To test whether the two starting elements change the meaning of MCCs, we compared responses to playbacks of piping calls versus beg-pipe calls and peow-pipe calls. For the seven groups for which we performed both piping and beg-pipe playbacks, maternal contact calls were the primary response recorded. However, such responses were at least twice as frequent during piping playbacks than beg-pipe playbacks (Goodness of fit χ^2^ = 3.86, d.f. = 1, *P* = 0.05; Fig. 5A). Additionally, although the frequency of maternal contact calls responses were similar during piping and peow-pipe playbacks across the six groups in which we tested both (χ^2^ = 0.72, d.f. = 1, *P* = 0.39), only peow-pipe playbacks generated beg-squawk responses by dominant females (χ^2^ = 18.0, d.f. = 1, *P* < 0.001, Fig. 5B)—an overt maternal call used to induce allo-feeding (Table I). That playbacks of piping calls alone versus in-association with begging calls and peow elements altered responses suggests that those starting elements modify the meaning of piping calls, rather than attributing an entirely new meaning to the new sequence.

## Discussion

Maternal contact calls are produced exclusively by breeding females, with the core of such calls being a series of loud, high-pitched piping elements. While the core piping calls can occur alone, more than 80% of such calls are initiated by acoustically distinct elements from other calls. In the two such cases considered here, both acoustic analyses and playback experiments suggested that piping calls can be initiated by the first (peow) elements of middle-distance contact calls (forming peow-pipe calls) and by broadband adult begging calls (forming beg-pipe calls). Further, playback experiments also suggested that initiating piping calls with peow elements and begging calls modifies function, with responses by dominant females being exaggerated during peow-pipe playbacks and attenuated during beg-pipe playbacks, relative to piping call playbacks. Finally, neither peow nor begging elements were more individually specific than piping elements. This study adds to the handful of examples providing evidence for syntax-like structures in animal communication systems and provides potential insights into candidate forms and functions that may have characterized syntax at its outset in the hominin lineage.

In linguistics, syntax describes the rule-guided combination of meaningful sounds (e.g., morphemes and words) to form higher-order structures (e.g., phrases and sentences) (Berwick & Chomsky, 2019; Hurford, 2012, 2014). However, a plausible hypothesis is that such an advanced form of syntax had simple origins (Martins & Boeckx, 2020). One of the few means of shedding light on candidate origins of syntax is to investigate the form and function of syntax-like sound arrangements in animal communication systems (Collier *et al*., 2014; Suzuki & Zuberbühler, 2019; Townsend *et al*., 2018). Basally, syntax at least requires the linear combination of acoustically distinct, “meaning-bearing” units together into larger, more meaningful structures. Under this proposed minimalistic criterion approach, three key findings are therefore required to satisfy the definition of syntax-like structures in animal communication systems. First, at least one call must be used both alone and in-combination with another call or call element (Hurford, 2012; Suzuki & Zuberbühler, 2019). In support, we found that the acoustic properties of begging calls and peow elements from middle distance calls were equivalent when used in these contexts and in combination with piping calls. Furthermore, responses to playbacks of natural maternal contact calls in peow-pipe and beg-pipe combinations were equivalent to those in which we generated such maternal contact calls artificially using peow elements from middle-distance contact calls and begging elements from begging calls. These results suggest that maternal contact calls can include combinations of other calls or call elements. Second, at least one call used in-combination needs to carry specific meaning (Hurford, 2007, 2012; Suzuki & Zuberbühler, 2019). In support of this requirement, maternal contact calls, middle-distance (peow pee) calls, and begging calls are all used in different contexts (Crane *et al*., 2016; Table I). In this study, piping call playbacks largely induced maternal contact call responses by mothers, playbacks of middle-distance contact calls were largely met with middle- and long-distance contact calls by group members, and begging calls typically failed to induce vocal responses. Finally, to qualify as syntax-like, calls used in combination need to modify meaning (Hurford, 2007, 2012; Suzuki & Zuberbühler, 2019). Again in support, playbacks of maternal contact calls in which piping elements were preceded by peow elements (peow-pipes) led mainly to beg-squawk responses, as opposed to maternal contact call responses during piping call playbacks, whereas beg-pipe playbacks typically failed to generate a vocal response.

Perhaps the simplest means of generating syntactic-like call combinations is through predicate argument type processes. For example, in both banded mongooses (Jansen *et al*., 2012) and chimpanzees (*Pan troglodytes schweinfurthii*, Leroux *et al*., 2021), individually diagnostic call structures are appended to other calls seemingly to encode individual identity. Our evidence that this predicate argument-like construction can account for combinatoriality in maternal contact calls of chestnut-crowned babblers is not compelling. For example, in the two examples above, a single, otherwise meaningless signature is appended to multiple functionally distinct calls, but in babblers two acoustically distinct call elements are appended to another functional call. In addition, neither begging nor peow elements are more individually specific than piping elements. Although it is conceivable that adding two acoustically distinct calls together will increase discernibility of individuals, it seems unlikely that this is the primary function of combinatoriality in the chestnut-crowned babbler system.

A second alternative is that combinatoriality is a product of affixation type processes, as first suggested in Campbell’s monkeys (Ouattara *et al*., 2009). In this species, the addition of “oo” sounds after predator (e.g., “kraak”) alarm calls generalizes the referential specificity of the threat (Coye et al., 2015; Ouattara *et al*., 2009; Schlenker *et al*., 2014, 2016). In chestnut-crowned babblers, peow elements are, similarly to “oo” sounds, also contained in another, functionally distinct call but not in isolation (in contrast to the begging call), in line with a potential affixation-like function. That peow elements exist in middle-distance contact calls, but when “prefixing” piping elements changes maternal vocal responses, has ostensible similarities with this Campbell’s monkey study. One possibility is therefore that peow elements modify the meaning of both pee elements (in middle distance contact calls) and piping elements in peow-pipe maternal contact calls, despite pee elements not being used in isolation. Unfortunately, we did not perform peow or pee only playbacks to test whether either carries meaning in isolation. Further work is required to clarify whether peow elements operate as affixes or carry independent meaning.

A final alternative is that our results are consistent with rudimentary idiomatic compounds and/or conjunction type processes. Our current vision of these two processes in animals come from a handful of species. In putty-nosed monkeys, two alarm calls can be combined, neither of which induce movement in isolation, but do so in combination, which has been suggested to be consistent with idiomatic compounds—where a qualitatively new meaning is generated from a combination call (Arnold & Zuberbühler, 2006). By contrast, in pied babblers (Engesser *et al*., 2016) and Japanese tits (Suzuki *et al*., 2016) alarm and recruitment calls are combined (in this order) to generate group-level mobbing of putative predators, which are more consistent with basic conjunction-like processes. However, the degree to which responses to call combinations need to differ from those induced by their constituent parts to conform to one or other of these two hypotheses is not clear, and it might be that idiomatic- and conjunction-like processes are not wholly independent in animals. Although it is clear in our study that playbacks of piping calls, beg-pipes and peow-pipes all generated different vocal responses by females, whether they were sufficiently different to qualify as idiomatic is ambiguous. For example, playbacks of beg-pipes significantly reduced interest by the dominant female, relative to playbacks of piping alone, whereas playbacks of peow-pipes generated more overt vocal responses by the dominant female. Studies investigating more subtle changes in behavior by group members in response to playbacks of the dominant female in their group following her temporary removal are likely to be necessary to tease these potential mechanisms apart.

In conclusion, we provide further evidence for syntactic-like structuring in nonhuman animals by demonstrating that chestnut-crowned babblers flexibly recombine components of at least three calls together into larger call combinations. Whilst follow-up perceptual work is central to further elucidate the precise semantic relationship between the individual calls and the resultant combinations as well as the role call order plays (Suzuki *et al*., 2016), this work is consistent with previous findings suggesting compositionality as a communicative mechanism in the combinatorial structures of animals. The evolutionary implications of this growing body of data are manifold, but most pertinently these data suggest that arrangements of individual calls into simple structured combinations may well be a key initial step characterizing the emergence of more complex, hierarchical syntactic systems, including language. Further studies are required to elucidate the full means by which animals combine vocal structures and how such structure provide proto analogues to human syntax.

## Data Availability

All data needed to evaluate the conclusions of this study are available from the Open Science Framework (Spiess *et al*., 2022).
